# Compact Hip-Force Sensor for a Gait-Assistance Exoskeleton System

**DOI:** 10.3390/s18020566

**Published:** 2018-02-13

**Authors:** Hyundo Choi, Keehong Seo, Seungyong Hyung, Youngbo Shim, Soo-Chul Lim

**Affiliations:** 1 Device & System Research Center, Samsung Advanced Institute of Technology, 130 Samsung-ro, Yeongtong-gu, Suwon-si, Gyeonggi-do 443-803, Korea; hyundo.choi@samsung.com (H.C.); keehong.seo@samsung.com (K.S.); seungyong.hyung@samsung.com (S.H.); ddalbo.shim@samsung.com (Y.S.); 2 Department of Mechanical, Robotics and Energy Engineering, Dongguk University, 30, Pildong-ro 1gil, Jung-gu, Seoul 04620, Korea

**Keywords:** hip force sensor, hip exoskeleton, gait assistance, force sensor, wearable sensor, rehabilitation robotics

## Abstract

In this paper, we propose a compact force sensor system for a hip-mounted exoskeleton for seniors with difficulties in walking due to muscle weakness. It senses and monitors the delivered force and power of the exoskeleton for motion control and taking urgent safety action. Two FSR (force-sensitive resistors) sensors are used to measure the assistance force when the user is walking. The sensor system directly measures the interaction force between the exoskeleton and the lower limb of the user instead of a previously reported force-sensing method, which estimated the hip assistance force from the current of the motor and lookup tables. Furthermore, the sensor system has the advantage of generating torque in the walking-assistant actuator based on directly measuring the hip-assistance force. Thus, the gait-assistance exoskeleton system can control the delivered power and torque to the user. The force sensing structure is designed to decouple the force caused by hip motion from other directional forces to the sensor so as to only measure that force. We confirmed that the hip-assistance force could be measured with the proposed prototype compact force sensor attached to a thigh frame through an experiment with a real system.

## 1. Introduction

With aging, muscle mass tends to decline, which can be related to diminishing strength and musculoskeletal function [1]. Aging is also related to significant changes in bones and joints, including loss of bone mass and density and/or skeletal geometry change in the area of the hip. Hip fractures can be caused by osteoporosis and these will increase as a result of population aging, possibly reaching 2.6 million by 2025 and 4.5 million by 2050 [2]. Aging can also cause mobility limitation and the incidence and prevalence of gait disorders are high in communities of older adults and are associated with a greater risk of institutionalization and death [3].

Recently, many exoskeleton systems have been proposed to provide a convenient way for helping the mobility of elder persons. These exoskeleton devices can help aging seniors strengthen their weakened muscle and bone function and can reduce cases of falls and catastrophic injuries [4]. Especially, lower limb exoskeleton systems and control strategies have been proposed to provide walking convenience and to assist mobility by supporting the motion of the lower limbs and increasing muscle power while being worn. One interesting study to reduce metabolic cost with unpowered exoskeletons was by Collins et al. [5] who reduced the energy cost without providing an additional energy source. The device used a clutch to sustain a force passively and delivered no net positive mechanical work. In their experiments, the metabolic cost of walking was reduced by 7.2 ± 2.6% for healthy people with a walking pattern comprising large strides. Moreover, many systems with a lower limb exoskeleton are focused on adding external energy using an actuator to make the exoskeleton become a personal walking assistant [6,7,8,9,10,11,12].

To guarantee power assistance efficiency and walking safety, the assistance force or torque of powered exoskeleton systems should be adaptable to the user’s walking style [13] and neural oscillators have been used in a number of studies to synchronize the motion between the human and robot trajectories [14,15,16]. Most developed exoskeleton systems for lower limbs are based on gait estimation with joint torque needed to perform movement [6,8]. The torque from the muscle of the user can be estimated by measuring the applied external torque at each exoskeleton joint [17] and by removing the external torque of the user’s motion such as inertial, Coriolis and gravitational torque. In some studies, the user’s posture is given by joint angles while motion intention is estimated based on an electromyographic (EMG) pattern of the user’s muscles [18,19]. In another research, EMG and an extended physiological proprioception-integrated interface to the exoskeleton was developed for harmonious control of the exoskeleton [20].

However, these exoskeleton systems have some limitations when implementing them for daily-life usage. One is the complex subject- and session-dependent calibrations that are required to calibrate users’ variations in body type, age and race and much of the anthropometric data provided by previous research may not match an actual user because of each person’s variations [21,22,23]. The differences between the properties of subjects, such as length of leg, size and shape of bones, deformation cartilages and soft tissues surrounding bones and locomotion type, affect the accuracy of the kinematic model [24]. As the kinematics depends on the variation of the user, it is very difficult to predict the torque of the gait assistant exoskeleton system using the kinematic model. Another limitation is the size of an exoskeleton system; most prototypes tend to be too heavy and bulky for use in daily-life movement assistance, so elderly people may not be able to fit an exoskeleton to their daily lives. Human joint torque, joint angle, movement acceleration and body tilting are used as measures to sense the intention of a user but sensors for these actions are bulky and their attachment structures are complicated, thus they are restricted to applications in which these factors and the cost take elderly persons into account.

To assist lower limb motion and overcome the mentioned limitations, we developed various kinds of compact and lightweight exoskeletons for elderly people in our previously reported studies [13,25,26,27]. The exoskeletons included partially assisting devices for only the hip and ankle and a fully assisting device for all of the joints in the lower limbs. Some studies have shown that exoskeletons can be effectively controlled by measuring the interaction force [28,29]. In our previous system, we used potentiometers and encoders for sensing the user’s motion and supporting and assisting torque was given to the user from the calculated model because the system was not able to measure the interaction force between the user and the exoskeleton directly. The delivered torque was estimated from the current in the motor via parameters and modeling of the motor and the reduction gears. However, the lubrication state in the gears was different as time passed and the force of the actuator was sometimes quite different when the model parameters were changed. In this case, in order to deliver the necessary power and torque exactly, we needed to calibrate the actuation module and establish another look-up table.

More robust and stable torque sensing is needed to monitor and control the assisting torque of the exoskeleton. In the proposed sensor system, the exoskeleton needs to respond to sudden actions by the user or in response to an external disturbance. Furthermore, the sensor must monitor the assistance torque state and immediately sense an abnormal torque state of the gait-assistance device. Even though the merit of current force sensors is sufficient for the stable operation of exoskeleton devices and ensures their safety for users, their use is currently limited due to their size and cost in conventional systems.

In this paper, we propose a novel compact and lightweight hip torque sensor that measures interaction force to implement a compact hip exoskeleton during various gait tasks such as standing, level walking, stair ascent and stair decent. The proposed sensor system was easily implemented to the existing exoskeleton by replacing the frame with the sensor-embedded frame. The sensor has a simple structure and its thickness is under 5 mm. It only senses the torque of the hip joint with two force sensitive resistors and does not respond to undesired parasitic force and torque. The sensor provides interaction torque data between the user and the hip exoskeleton. It is practically useful since the sensor only requires two analog-to-digital convertors for torque sensing.

This rest of this paper is organized as follows. In Section 2, we describe the configuration of the sensor hardware for hip exoskeleton system and how to measure hip force and performance evaluation and includes modeling of the sensor system. Section 3 is concerned with the implementation of force sensor for gait assistance. We finally draw conclusions and note the scope for further study in Section 4.

## 2. A Hip-Force Sensor for a Gait-Enhancing Mechatronic System

### 2.1. Autonomous Hip Exoskeleton with a Thin-Force Sensor

**Figure 1:**
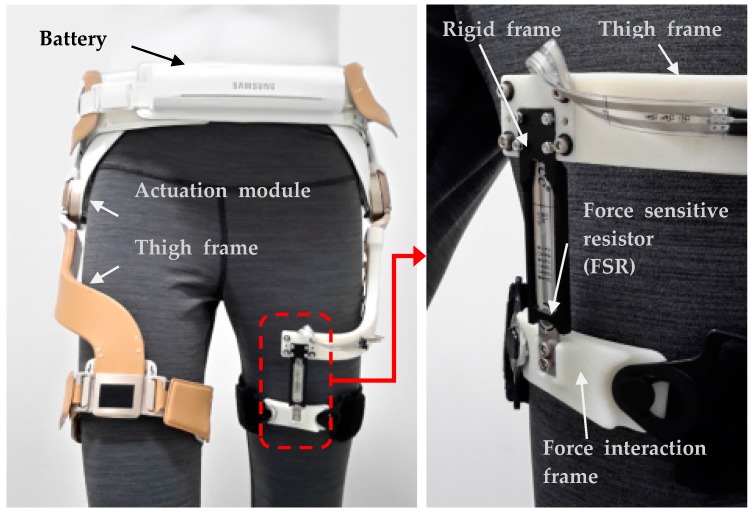
Autonomous hip exoskeleton for gait enhancement and thin assistance torque sensor.

We developed a prototype of the gait-assistance device to assist individuals with walking disabilities and called it GEMS (Gait-Enhancing Mechatronic System), as shown in Figure 1. Clinical results using the earlier version of GEMS indicated that the robot improved the spatiotemporal gait characteristics and metabolic rates of elderly individuals [30]. It also demonstrated positive results with respect to stroke [31] and Charcot-Marie-Tooth [32] patients.

The device is designed to deliver assistance torque in the extension and flexion of the hip joints. The robot is worn around the waist and thighs. The exoskeleton consists of a pair of actuators for the left and right hip joints, a hip brace around the waist, a pair of thigh frames that transmit assistance torque from the actuators to the thighs and fabric belts at the ends of the thigh frames. Two 70 W motors (one at each hip joint) provide torque to the joints in the sagittal plane (i.e., flexion and extension). The medial-lateral direction at the hip joint is passive to permit free movement. GEMS is “autonomous” because it carries all of its electronics, computations, actuations and power sources on the device. The total weight of the device is 2.4 kg with a maximum torque of 12 Nm at each joint. The battery is positioned in the front belt while the CPU is positioned in the backpack to distribute the weight imposed on the user evenly. The operating time is approximately 2 h. There are two joint angle sensors in the actuator modules and there is an inertial measurement unit in the backpack [33].

The proposed hip force sensor is shown in the right of Figure 1. It measures the interaction force between lower limb and exoskeleton system which is designed to deliver assistance torque to extension and flexion. Our previous prototypes measured the hip joint angle for gait recognition and the delivered torque was estimated from the current in the motor, parameters and modeling of the motor and the reduction gears. For more accurate measuring of the interaction force between the hip exoskeleton system and the generated output torque, we developed and attached the hip-force measurement sensor system designed by attaching two force-sensing resistors (FSRs) to measure the *z*-axis force. An FSR was attached to both surfaces of the center plate in the developed sensor system structure. This sensor system is able to sense the normal force to the sensor while decoupling other directional forces. With this sensor design, the vertical force to the *z*-axis does not give any resistance variation to the sensor. Furthermore, the FSRs were selected due to other advantages that they offer such as being in the form of a film and so are thin.

### 2.2. Design and Modeling of the Hip Force Sensor

**Figure 2:**
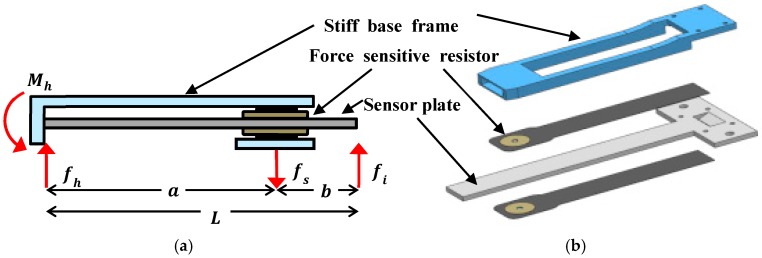
(**a**) Modeling of the assistance force sensor and (**b**) the structure of the sensor.

Figure 2 shows the structure and cross-section of the sensor system for the hip exoskeleton. The stiff base frame and sensor plate are rigidly connected to one end and an FSR is attached to both side of the sensor plate. When the interacting assistance force ($f_{i}$) between the hip exoskeleton and user is applied to the sensor plate, the normal sensing force ($f_{s}$) is applied to the FSRs because the sensor plate transfers the force to them, thus we can measure the reaction force of assistance force from the FSRs on both sides of the sensor plate. Furthermore, the sensor system causes no normal force on the FSR sensor when horizontal force is applied. Instead, the horizontal force would be transferred to the stiff base frame through the sensor plate.

The proposed force sensor measures the interacting assistance force ($f_{i}$) between the hip exoskeleton and the user to control or monitor the assistance force. Here, we determine how the normal sensing force ($f_{s}$) is related to the interacting assistance force ($f_{i}$). The sensor structure is statically indeterminate because the external force ($f_{i}$) causes three reactions and the number of unknowns is more than the number of static equations. When the external force ($f_{i}$) is applied to the structure, three reactions need to be calculated for the sensing relationship. We calculate deflections of the single-ended beam by the interacting assistance force ($f_{i}$) and the normal sensing force ($f_{s}$). On the FSR sensing area, the deflection of the sensor plate by each force can be written as

(1) $${\left(\delta_{s}\right)}_{i}=\frac{f_{i}a^{2}}{6EI}\left(3L-a\right)$$

(2) $${\left(\delta_{s}\right)}_{s}=\frac{f_{s}a^{2}}{3EI}2a$$

where ${\left(\delta_{s}\right)}_{i}$ and ${\left(\delta_{s}\right)}_{s}$ are deflections of the sensor plate on the sensing area by $f_{i}$ and $f_{s}$, respectively; EI is the flexural rigidity of the sensor plate; L is the length of the sensing plate; and a is the distance of sensing area from the connection point.

Now we can find the original deflections in the sensor plate ($\delta_{s}$) by superimposing the separate deflections caused by the forces. Since the original deflection of the sensor plate on the FSR is zero, it yields $\delta_{s}={\left(\delta_{s}\right)}_{i}-{\left(\delta_{s}\right)}_{s}=0$. Subsequently, we can obtain the relationship between the normal sensing force ($f_{s}$) and the interacting assistance force ($f_{i}$) as follows:

(3) $$f_{i}=\frac{2a}{6L-a}f_{s}$$

Equation (3) means that the force acting on the sensing area is determined by the interacting assistance force and the geometric parameters of the sensor. The normal force ($f_{s}$) acting on the FSR has a linear relationship with the interacting assistance force ($f_{i}$). Furthermore, the fixed end of the sensor can be replaced by a pivotal joint. In this case, we can obtain another relationship between the normal sensing force ($f_{s}$) and the interacting assistance force ($f_{i}$) by taking a moment equilibrium equation about the free pivot. The following equation shows that the forces in the pivot-end are proportional to each other:

(4) $$f_{i}=\frac{a}{L}f_{s}$$

In the present study, we used a statically undetermined fixed-end structure because the pivotal revolute joint could have made the sensor thick in order to support a high horizontal force. The normal sensing force ($f_{s}$) in Equation (4) is sensed by the FSR sensor on both sides with each FSR sensing compressional force only. Since the electrical resistance of the FSR is known to be inversely proportional to the normal sensing force ($f_{s}$), the relationship can be written as

(5) $$f_{s}=k/R$$

where k is a constant and R is the resistance of the FSR. Using a voltage divider followed by an operational amplifier, we can estimate the flexion and extension force of the hip exoskeleton by measuring the resistance of the FSR. The overall sensing performance depends on FSR characteristics, which because it is a kind of conductive polymer, has non-linear characteristics such as hysteresis, stress relaxation and creep [34]. This means that the sensor cannot hold a constant reaction force with the same strain and cannot maintain a constant strain with the same pressure and so multiple measurements performed under the same conditions can lead to different results. In the case of the strain gauge-based force sensor, the strain gauge has a specific value when no force is being applied to the sensor and it can be attached to elastic materials such as steel or aluminum, which have small drift and hysteresis. However, FSRs are compressed directly to induce resistance change, which is why their non-linear characteristics can be problematic when they are being used as force sensors.

Nevertheless, the proposed sensor structure can increase repeatability around the low force area. The FSR sensor has a tendency to have good repeatability when the resistance is high, which can be explained by variation of the parameters in Equation (5) as

(6) $$\partial f_{s}=-\frac{k}{R^{2}\partial R}.$$

Equation (6) shows that the uncertainty of the resistance does not have a large effect on the variation of the normal sensing force ($f_{s}$) when the resistance value is high. In other words, the sensitivity of the resistance to the sensing force is very low when the resistance is high and the applied force is low. Moreover, one of the important requirements for force sensors when used for force or torque control is to maintain the zero value stably when the external force is released, otherwise chattering usually happens in practical use and the system is constantly accelerated by the actuator when maintaining zero force control. The use of two separate FSRs can avoid the hysteresis and creep problems and the FSRs are operated in an area where the repeatability is better and they are always able to converge on the zero point when the applied force is released. Extension and flexion force are measured by a different FSR sensor.

Another feature of the proposed force is a pressure plate design for uniform pressure. By the contact state, the sensor has different output value [35]. Flat surfaces produce highly accurate results on multiple sensing occasions and the output of the sensors also changes depending on the underlying surface. Hence, the pressure plate repeatedly makes the same contact state between the frame and the FSRs.

### 2.3. Calibration of the Force Sensor and Measuring Hip Force

**Figure 3:**
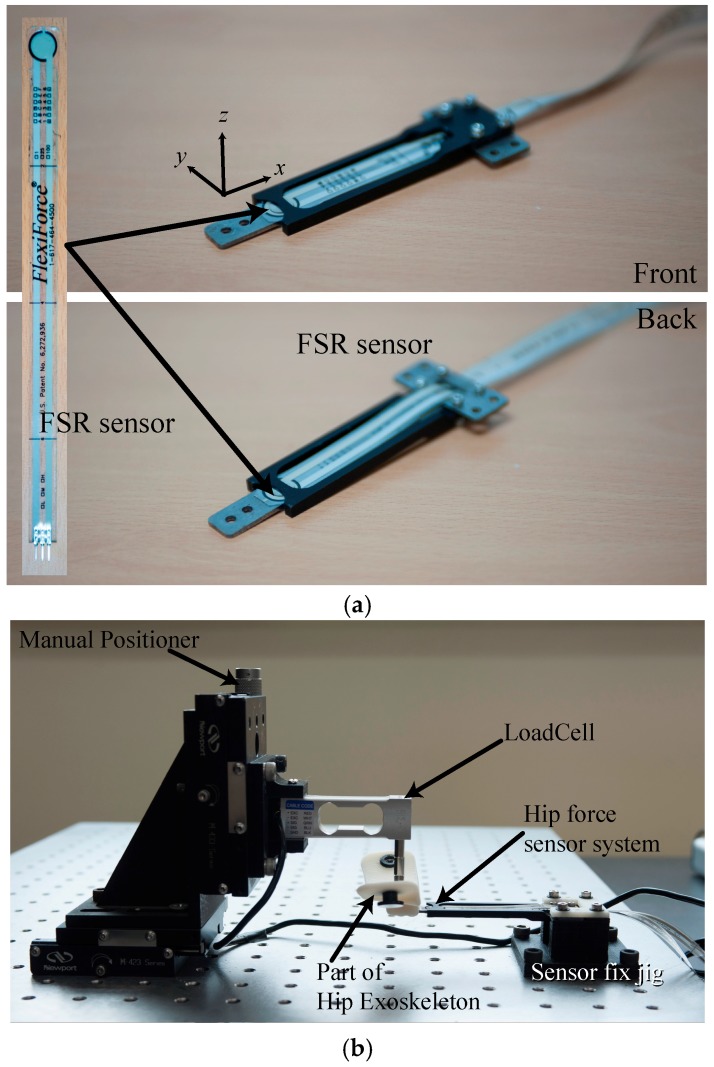
(**a**) The developed sensor system and (**b**) force measurement and calibration system setup.

The experimental setup shown in Figure 3 was developed to measure the reflection of the hip force to the exoskeleton system. The change in the sensed voltage from the Wheatstone Bridge of the two FSR sensors and the additional two resistances was measured with an AD converter (NI USB-6343, National Instruments, Austin, TX, USA) during which a force was provided to the hip force sensing part in the exoskeleton system using a load cell (BCL-3L, CAS, Seongnam, Korea). Three axis precision stages with a manual positioner that was able to move the sensing part and load cell with a precision of <1 μm were used to apply force to the sensing part with 1 mN resolution. The sensing part contacted the tip of the load cell and force was applied to it in each direction and at each position. Figure 3b shows the *z*-axis force measurement setup. Dynamic testing was performed using the measurement setup consisting of repeatedly loading and unloading the sensor 4 times. Identical measurements were performed for force applied to the *z*-, *y*- and *x*-axes.

**Figure 4:**
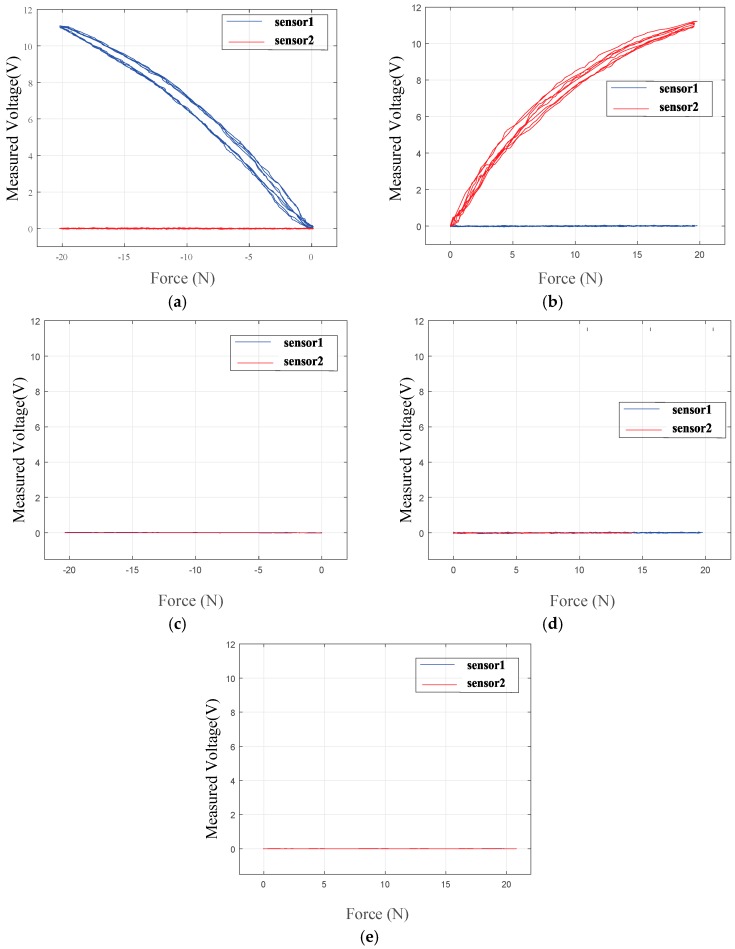
Experimental results of the relationship between the input force on the *z*-axis (**a**,**b**), the *y*-axis (**c**,**d**) and *x*-axis (**e**) and the voltage variation of each sensor.

Figure 4 shows the experimental results of the relationship between the input force and the voltage variation of each sensor. Figure 4a,b shows graphs of the relationship between the loading force on the *z*-axis and the measured voltage at the two sensors. For this system’s structural characteristics, variation output occurred in one sensor when applying force to the system while the other sensor did not register any change. During the loading stage, the sensors followed the upper curve, while during the unloading stage, they followed the lower side of the curve. From previous research, the calibration of the FSR sensor for static and dynamic applications was shown to compensate for this hysteresis [36]. Figure 4c–e shows graphs of the relationship between the loading force on the *y*- and *x*-axes and the measured voltage at the two sensors. These graphs indicate decoupling in each direction, which indicates that the developed exoskeleton system with two FSR sensors was able to read the exerted force from the knee/hip to the system without interference by the other force. The sensing accuracy is 1.5 N due to the nonlinearity of the FSR sensor and hysteresis of the sensor. Previous research reported that the Weber fraction of force is higher than 7% [37,38,39]. The sensing accuracy is below the Weber fraction, which is allowable for a wearable device, because a human cannot discriminate the force difference.

## 3. Implementation of the Force Sensor in the Gait-Assistance Exoskeleton System

### 3.1. Control of the Gait-Assistance Exoskeleton System

We developed an assist-as-needed algorithm for the assistance system consisting of two main functional modules for gait-phase estimation and assistance-torque computation. The gait phase was determined by adaptive oscillators (AOs) which were able to estimate the frequency of periodic input continuously and instantaneously as they learned the input signal on-line. Furthermore, a modified version of the AOs, particularly shaped adaptive oscillators (PSAOs) were able to detect the gait phase and frequency. This implementation for gait assistance was first demonstrated in [27].

A PSAO is in fact a group of oscillators that *learns* input θ as a linear combination $\hat{\theta }$ of periodic basis functions f and sine functions

(7) $$\hat{\theta }=\alpha_{0}+\alpha_{1}f\left(\phi_{1}\right)+\overset{n}{\underset{i=2}{\sum }}\alpha_{i}\sin\phi_{i}$$

where $\alpha_{i}$ and $\phi_{i}$ are the amplitude and phase of the *i*th oscillator, respectively; and $\alpha_{0}$ is a DC bias term. The learning error $e=\theta -\hat{\theta }$ updates phase $\phi_{i}$ and amplitude $\alpha_{i}$ of each oscillator to reduce the error e to zero for i≥2:

(8) $${\dot{\phi }}_{i}=i\omega +k_{\phi }e\cos\phi_{i}$$

(9) $${\dot{\alpha }}_{i}=k_{\alpha }e\sin\phi_{i}$$

From Equation (8), the frequency of the *i*th oscillator is around iω, which is an integer multiple of ω. The base frequency ω is the frequency of the first oscillator updated by

(10) $$\dot{\omega }=k_{\omega }eg\left(\phi_{1}\right)$$

where g is the partial derivative of f(ϕ) with respect to ϕ. Other states of the first oscillator are updated by

(11) $${\dot{\phi }}_{1}=\omega +k_{\phi }eg(\phi_{1})$$

(12) $${\dot{\alpha }}_{1}=k_{\phi }ef(\phi_{1})$$

Learning error e also updates the DC bias term:

(13) $${\dot{\alpha }}_{0}=k_{0}e$$

For application to hip joint assistance, input θ can be the hip joint angle. If gains of k with subscripts of ϕ, 0 and α are properly selected and if the input is nearly periodic, then $\hat{\theta }$ asymptotically converges to the input θ and the phase $\phi_{1}$ of the first oscillator-estimated gait phase after scaling 2π down to 1. Base frequency ω also represents the gait frequency in rad/s.

In addition, the assistance torque is determined by looking up a table of torque profiles as a function of gait phase and walking speed using the method described in [17]. Inspired by biological torque patterns in the literature, the assistance torque profile used in this study was designed to have an extension peak and a flexion peak. The torque profile for low speed had lower torque peaks. The walking speed was estimated via an inertial measurement unit on the pelvis using linear regression [27].

**Figure 5:**
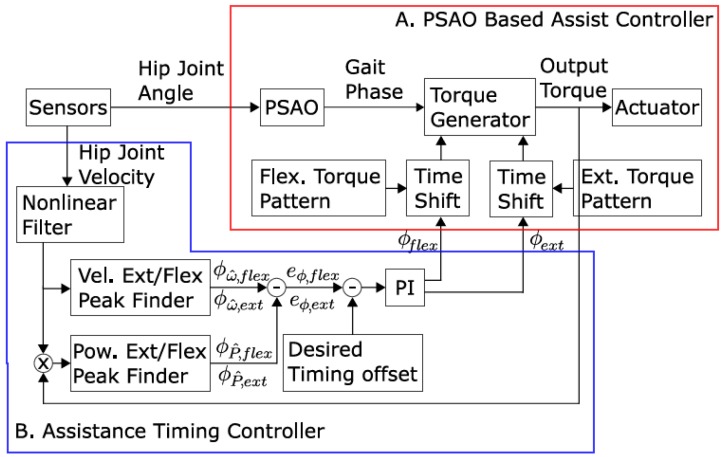
Assistance controller architecture.

We were able to adjust the assistance timing for each subject manually in consideration of inter-subject variance in gait. The adjustments were made iteratively until the subject was content. We supposed that the subjective sense of assistance timing originates from the relative timing between human motion and assistive action and as variables for these, we selected the peak timing of hip joint velocity for flexion and the peak timing of actuator power corresponding to the flexion, respectively. Timings were defined in terms of gait phase within a gait cycle. Once the target phase difference between the peak joint velocity and the peak assistance power was registered for a subject, phase offsets for assistance torque were autonomously controlled to keep the phase difference close to the target value. To reduce the control complexity, the extension power peak was controlled to coincide with the flexion power peak of assistance on the opposite side. A recent study revealed that for healthy subjects, the metabolic cost of walking was most reduced when the flexion power peak was around 0% to 4% of the gait cycle past the flexion velocity peak [40]. The overall control architecture for the assistance-timing controller along with phase detection and torque generation is illustrated in Figure 5.

### 3.2. Force Sensor Test Results

**Figure 6:**
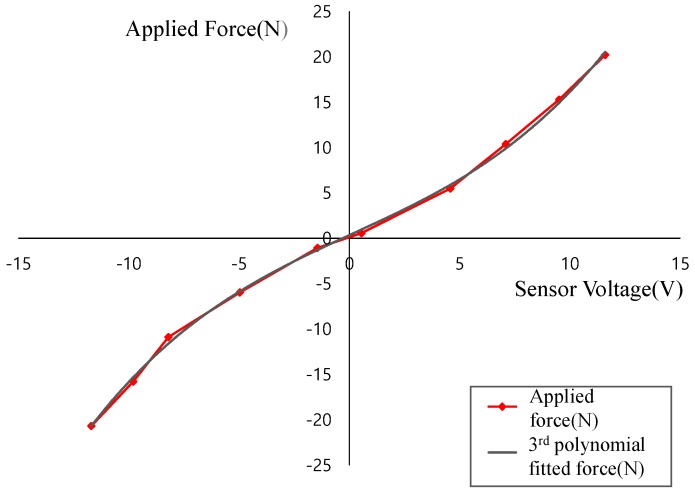
Relation between applied force and sensor voltage.

To assess the feasibility of the proposed force sensor in our target application (a wearable assistive robot), we replaced one of the thigh frames with the proposed sensor to measure the interaction force. The sensor features a mechanically connecting thigh fastening module and an actuation module and it transfers mechanical energy between them. We calibrated the sensor with the whole hip assistance device, because the sensor needs to hold the additional mass of the fastening module. The sensing voltage is measured four times with each measurement force, as shown in Figure 6.

**Figure 7:**
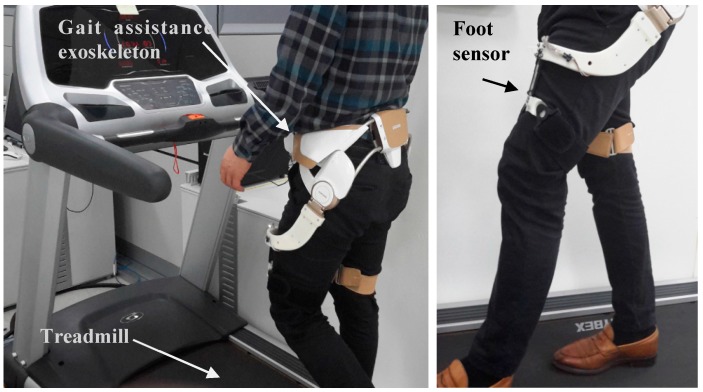
Experimental setup with a treadmill.

**Figure 8:**
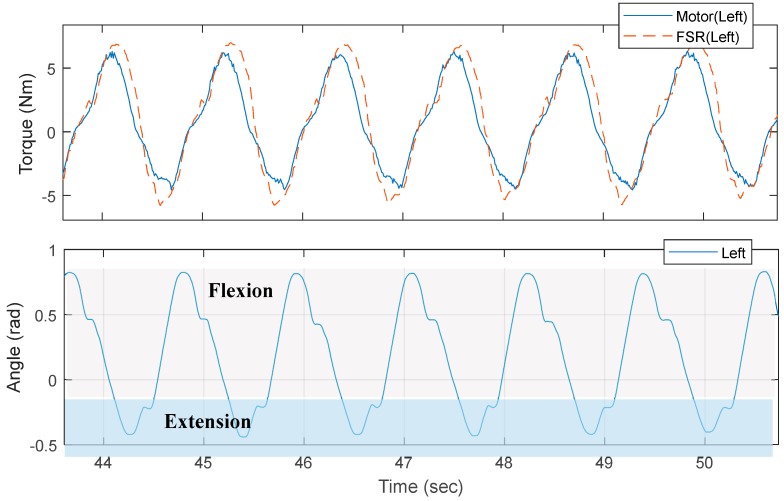
Assistive torque estimated from the force-sensitive resistor (FSR) sensor and motor current for comparison. The hip joint angle while walking is also presented.

The sensor test was performed at a fixed speed (3 km/h) for one subject for several minutes on a treadmill, as shown Figure 7. Excerpt data from the test are shown in Figure 8. The assistive torque was estimated using the FSR sensor in the following manner. The third order polynomial fitting (Figure 6) for each direction of force was found after which the net force was computed as the sum of forces in each direction. Next, the distance from the joint to the pressure point was projected onto the sagittal plane of the joint and measured and finally, the torque was computed by multiplying the projected distance and the net force. This procedure was only used for the preliminary study for the feasibility of our FSR sensor, thus scaling factors for the FSR might not have been accurate due to errors in fitting and projection. However, from the plot in Figure 8, there is an obvious difference that cannot be fitted by scaling factors and we attributed such a difference to the friction and inertia in the actuator as well as to the elasticity in the thigh frame.

The measured current is proportional to the motor torque of the device. The current can indicate the interaction force between the device and the user. However, it could not measure the exact torque or force that the user might feel. The interaction force is different from the estimated force from the current because of the presence of nonlinearities [41,42,43]. The interaction force between the device and the user is transmitted through the fastening part, the thigh frame, the gear-train and the motor sequentially or reverse sequentially. The presence of nonlinearities, such as friction [44] and backlash hysteresis of gear system [45], limits its performances for controlling system by measuring current. In the absence of a transmission model that accounts for these nonlinear behaviors [43,46], also have effect on poor performance of controlling the system by measuring current. The interaction force can be dedicated to the inertial force change of the components and to overcome gravitational force. Additionally, it can be dissipated by the friction and damping of the systems. However, the proposed sensor is placed in the front-end of the thigh fastening module where the force interacts with the user. The force from the proposed force sensor could be closer to the force the user feels, because the interaction force is transmitted directly to the sensor.

## 4. Conclusions

In this paper, we propose a compact force-sensor system for a hip-mounted exoskeleton for seniors comprising an FSR on both sides of a sensor plate to measure the provided force from hip motion. The FSR sensor measures flexion and extension force of the hip joint caused by hip motion only. Lateral and longitudinal forces are supported by the sensor frame structure while the assistance forces are transferred to the FSR sensors. We experimentally confirmed the feasibility of the developed FSR sensor in the exoskeleton system, the results of which are shown in Figure 7.

We are currently working on implementing the sensor with the assisted walking movements of various users and are also planning to implement force control such as admittance control and impedance control in order to provide more transparent force assistance and reduce motion resistance by the exoskeleton for the users. We believe that the developed system makes it possible to achieve natural assistance by driving the appropriate assistive force.
